# Effect of Low Temperature on Chlorophyll Biosynthesis and Chloroplast Biogenesis of Rice Seedlings during Greening

**DOI:** 10.3390/ijms21041390

**Published:** 2020-02-19

**Authors:** Yuqing Zhao, Qiaohong Han, Chunbang Ding, Yan Huang, Jinqiu Liao, Tao Chen, Shiling Feng, Lijun Zhou, Zhongwei Zhang, Yanger Chen, Shu Yuan, Ming Yuan

**Affiliations:** 1 College of Life Science, Sichuan Agricultural University, Ya’an 625014, China; yuqing@stu.sicau.edu.cn (Y.Z.); xiaoyangyang26@126.com (Q.H.); dcb@sicau.edu.cn (C.D.); shirley11hy@163.com (Y.H.); liaojinqiu630@sicau.edu.cn (J.L.); chentao293@163.com (T.C.); fengshilin@outlook.com (S.F.); zhoulijun@sicau.edu.cn (L.Z.); anty9826@163.com (Y.C.); 2 College of Resources, Sichuan Agricultural University, Chengdu 611130, China; zzwzhang@sicau.edu.cn (Z.Z.); roundtree@sohu.com (S.Y.)

**Keywords:** *Oryza sativa* L., chilling stress, chlorophyll biosynthesis, chloroplast biogenesis, epidermal characteristics

## Abstract

Rice (*Oryza sativa* L.) frequently suffers in late spring from severe damage due to cold spells, which causes the block of chlorophyll biosynthesis during early rice seedling greening. However, the inhibitory mechanism by which this occurs is still unclear. To explore the responsive mechanism of rice seedlings to low temperatures during greening, the effects of chilling stress on chlorophyll biosynthesis and plastid development were studied in rice seedlings. Chlorophyll biosynthesis was obviously inhibited and chlorophyll accumulation declined under low temperatures during greening. The decrease in chlorophyll synthesis was due to the inhibited synthesis of δ-aminolevulinic acid (ALA) and the suppression of conversion from protochlorophyllide (Pchlide) into chlorophylls (Chls). Meanwhile, the activities of glutamate-1-semialdehyde transaminase (GSA-AT), Mg-chelatase, and protochlorophyllide oxidoreductase (POR) were downregulated under low temperatures. Further investigations showed that chloroplasts at 18 °C had loose granum lamellae, while the thylakoid and lamellar structures of grana could hardly develop at 12 °C after 48 h of greening. Additionally, photosystem II (PSII) and photosystem I (PSI) proteins obviously declined in the stressed seedlings, to the point that the PSII and PSI proteins could hardly be detected after 48 h of greening at 12 °C. Furthermore, the accumulation of reactive oxygen species (ROS) and malondialdehyde (MDA) and cell death were all induced by low temperature. Chilling stress had no effect on the development of epidermis cells, but the stomata were smaller under chilling stress than those at 28 °C. Taken together, our study promotes more comprehensive understanding in that chilling could inhibit chlorophyll biosynthesis and cause oxidative damages during greening.

## 1. Introduction

Seedlings, growing in the darkness before emerging from the soil, undergo etiolation with long hypocotyls and closed cotyledons which contain undeveloped plastids called etioplasts that have no chlorophyll [1,2]. The greening process initiates when exposed to light as the seedlings come out of soil, and this de-etiolation process includes the reduction of hypocotyl elongation rate, cotyledon opening, chlorophyll synthesis, and chloroplast biogenesis, and subsequently, seedlings absorb light energy and transition to autotrophy [3,4].

Chlorophyll (Chl) has unique and essential roles in harvesting and transducing light energy in antenna systems, and charge separation and electron transport in reaction centers [5,6]. Chlorophyll level is an important index used to evaluate photosynthetic capacity. Chlorophyll content decreases under cold stress, which might be because low temperature suppresses chlorophyll biosynthesis, probably by inhibiting the activities of chlorophyll biosynthetic enzymes [7]. A previous study showed that the optimum temperature of divinyl reductase (DVR) activity was 30 °C, and low activity was observed at 10 °C and no activity was found at 50 °C [7].

Chls share a common biosynthetic pathway with other tetrapyrroles, including siroheme, heme, and phytochromobilin in plants, algae, and many bacteria [6,8,9]. Since tetrapyrrole intermediates are all photosensitizers that are easily activated by light, leading to highly toxic levels of reactive oxygen species (ROS) and photooxidative damage and growth retardation, Chl biosynthesis must be finely controlled to ensure healthy growth during the greening process [9,10]. Chl biosynthesis is a very complex process that is executed via a series of coordinated reactions catalyzed by numerous enzymes [11]. The process of Chl biosynthesis can be divided into three distinct phases. The first phase involves the synthesis of protoporphyrin IX (Proto IX) from glutamate [12]. The first committed precursor is *δ*-aminolevulinic acid (ALA), and its synthesis is a key control point in Chl biosynthesis. This step is catalyzed by glutamyl-tRNA reductase (GluTR), which is encoded by *HEMA* gene [8]. *δ*-Aminolevulinic acid dehydratase (ALAD) catalyzes aggregation of two ALA molecules into porphobilinogen (PBG). Four PBGs polymerize and further cyclize to yield uroporphyrinogen III (urogen III), when then decarboxylates to form coproporphyrinogen III (coprogen III). Coprogen III occurs via oxidative decarboxylation and oxygen-dependent aromatization reaction to form protoporphyrin IX (Proto IX) [9]. The second phase includes the synthesis of chlorophyll *a* (Chl*a*) from Proto IX. Proto IX is the branch point of the chlorophyll and heme biosynthetic pathways [6]. The insertion of Mg^2+^ into Proto IX forms Mg-protoporphyrin IX (Mg-proto IX), which is catalyzed by Mg-chelatase, but the insertion of Fe^2+^ into Proto IX catalyzed by ferrochelatase starts the heme branch [9]. Meanwhile, the photoreduction of Pchlide to chlorophyllide (Chlide), catalyzed by protochlorophyllide oxidoreductase (POR), is another important step. POR is the key enzyme in the light-dependent greening of higher plants [13]. Two POR proteins (PORA and PORB) and three POR proteins (PORA, PORB, and PORC) have been found in rice and *Arabidopsis*, respectively [14,15]. DVR catalyzes 3,8-divinyl-chlide *a* to yield chlorophyllide a (Chlide a), which further forms Chl *a* catalyzed by chlorophyll synthase (CHLG). The third phase is the interconversion of Chl *a* and chlorophyll *b* (Chl *b*) catalyzed by chlorophyllide a oxygenase (CAO) which is known as the chlorophyll cycle [16].

Chloroplasts are responsible for photosynthesis and the production of hormones and metabolites [17,18], which develop from proplastids that are present in the immature cells of plant meristems. Chloroplast biogenesis involves a coordinated function of plastid- and nuclear-encoded genes [19]. The process of chloroplast biogenesis accompanies the biosynthesis of chlorophylls, chloroplast proteins, carotenoids, and lipids, and the assembly of photosynthetic protein complexes including the light-harvesting complex (LHC), photosystem I (PSI), photosystem II (PSII), cytochrome *f/b_6_* (cyt*f/b_6_*), and adenosine triphosphate (ATP) synthase [1,17]. Many factors have direct impacts on chloroplast biogenesis, such as light, water, salt, leaf age, etc. [1,20].

Low temperature is one of the most severe weather events that destructively affect crop growth, quality, and yield [21,22]. Cold or chilling stress impairs the chloroplast microstructure, photosynthetic metabolism, and energy production [23], directly leading to inhibited photosynthesis, which is a severe threat to crop production. Additionally, the efficiency of photosynthetic electron transport in plants is significantly decreased under chilling stress, resulting in a burst of ROS that directly causes cellular oxidative damages and increases membrane rigidity [24,25,26,27]. Low temperature also disrupts the carbon reduction cycle and the control of stomatal conductance [28].

Rice (*Oryza sativa* L.) is a kind of thermophilic cereal crop that feeds almost half of the world’s population. Originating from tropical and subtropical areas, rice is highly sensitive to low temperatures, particularly during the greening process of the early seedling growth. In China, cold spells in late spring cause serious suppression of rice seed germination and young seedling growth, especially in the middle–lower Yangtze River region and southern China. In the past 30 years, 30%–50% rice seedlings suffered from chilling or cold stress, resulting in a reduction of 3–5 megatons every year [29].

Chlorophyll biosynthesis is seriously blocked by low temperatures during greening. However, the inhibitory mechanism by which this occurs is still unclear. Understanding how rice responds to low temperatures will provide valuable information and genetic resources for improving cold-stress tolerance. Therefore, the object of this study was to explore the response mechanism of chlorophyll biosynthesis and chloroplast biogenesis during the greening process under chilling stress and to provide valuable data for crop production.

## 2. Results

### 2.1. Effect of Chilling Stress on Plant Growth

**Figure 1:**
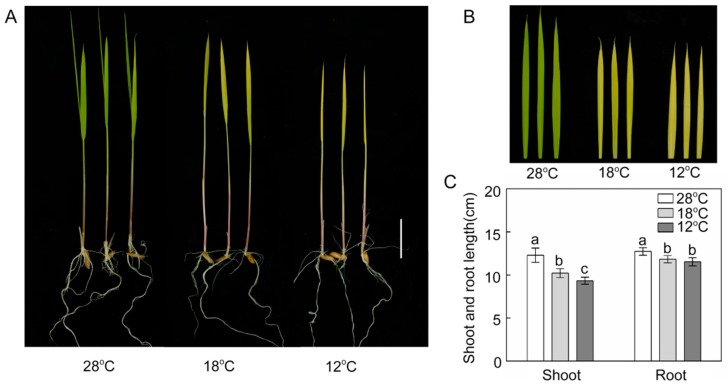
Effect of chilling stress on rice seedling growth (**A**,**B**) and shoot/root length (**C**) after 48 h of greening. Six day old etiolated seedlings were treated with 18 °C or 12 °C chilling stress. Date represent means ± SD of 10 replicate samples. Bars with different letters above the columns of figures indicate significant differences according to Duncan’s multiple range test at *p* < 0. Bar = 2 cm.

To investigate the effect of chilling stress on the growth of rice seedlings during greening, 6 day etiolated seedlings were treated with 28 °C, 18 °C, and 12 °C for 48 h in light (120 μmol m^−2^ s^−1^). After 48 h of greening, leaves were green and fully expanded at 28 °C (Figure 1A,B) and the leaves at 18 °C looked yellow-green and were fully expanded, while the leaves at 12 °C were yellow and incompletely expanded (Figure 1A,B). Compared with plants growing at 28 °C, chill-treated rice seedlings exhibited significantly lower shoot and root lengths after 48 h illumination (Figure 1C). These results showed that chilling stress significantly inhibited the greening process and the growth of rice seedlings.

### 2.2. Effect of Chilling Stress on Dry Weight, Protein, Chlorophyll, and Carotenoid Content

**Figure 2:**
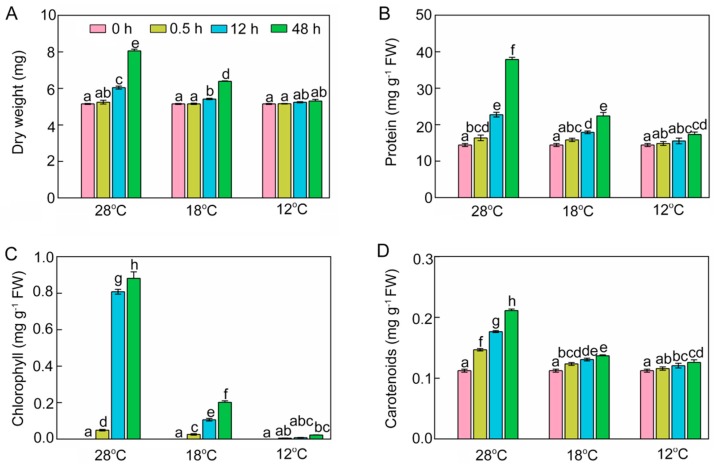
Dry weight (DW, **A**), soluble protein (**B**), chlorophyll (**C**), and carotenoids (**D**) of control (28 °C) and chill-stressed (18 °C and 12 °C) rice seedlings after 0 h, 0.5 h, 12 h, and 48 h greening. Six day old etiolated seedlings were treated with 18 °C or 12 °C cold stress. Seedlings were harvested at 0 h, 0.5 h, 12 h, and 48 h of greening and their DW, protein, chlorophyll, and carotenoid contents were measured. The error bars represent standard deviations of three independent biological replicates. Different letters indicate significantly different at *p* < 0.05 according to Duncan’s multiple range tests.

The dry weight (DW) of shoots was increased by 1.8%, 17.3%, and 56.5%, respectively, at 28 °C after 0.5 h, 12 h, and 48 h of light exposure (Figure 2A). However, the accumulation of dry matter was significantly inhibited under chilling stress. Shoot DWs were 10.3% and 20.7% lower after 12 h and 48 h at 18 °C compared with those at 28 °C. More severely, DWs at 12 °C were 13.2% and 34.1% lower after 12 h and 48 h of light exposure compared with those at 28 °C, and showed no significant change compared with the seedlings before the light exposure.

At 28 °C, protein contents of seedlings rose to 16.38, 22.74, and 37.90 mg·g^−1^ fresh weight (FW) after 0.5 h, 12 h, and 48 h of greening, respectively (Figure 2B). Low temperature reduced protein accumulation of rice seedlings during the greening process. There were only 22.41 mg·g^−1^ FW at 18 °C and 17.31 mg·g^−1^ FW at 12 °C after 48 h of light exposure.

Chlorophyll during the greening process was significantly increased at 28 °C. However, chlorophyll levels were 77.1% lower at 18 °C and 97.5% lower at 12 °C than at 28 °C (Figure 2C). Carotenoids at 28 °C increased to 0.21 mg·g^−1^ FW after 48 h of greening, while chill-stressed rice seedlings accumulated much lower levels of carotenoids (Figure 2D).

### 2.3. Effect of Chilling Stress on the Accumulation of Chlorophyll Intermediates in Rice Seedlings during Greening

To explore how chilling stress inhibits chlorophyll biosynthesis in rice seedlings during greening, the accumulation of chlorophyll intermediates was measured.

**Figure 3:**
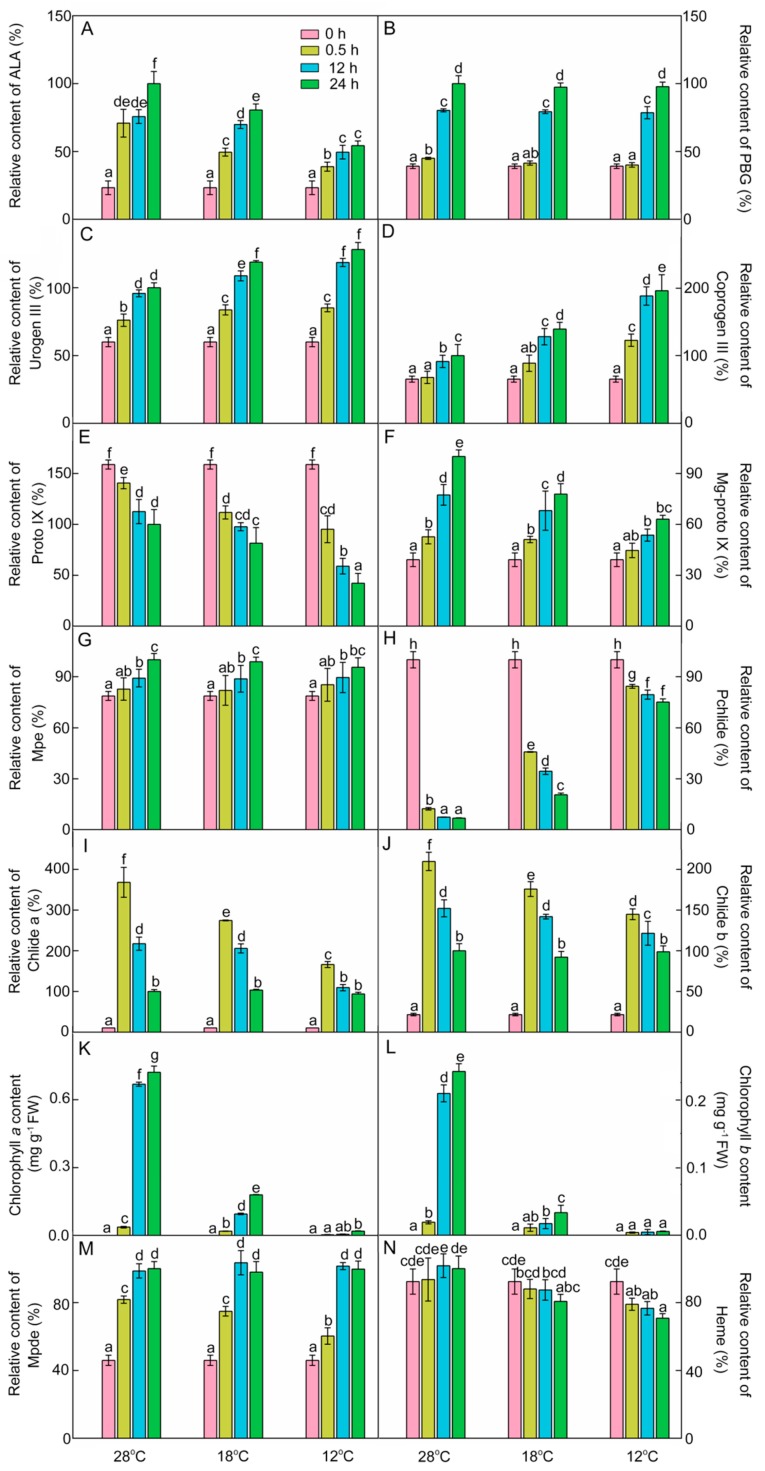
Chlorophyll biosynthesis intermediates during the greening period. *δ*-Amino levulinic acid (ALA, **A**), porphobilinogen (PBG, **B**), uroporphyrinogen III (urogen III, **C**), coproporphyrinogen III (coprogen III, **D**), protoporphyrin IX (Proto IX, **E**), Mg-protoporphyrin IX (Mg-proto IX, **F**), Mg-protoporphyrin monomethyl ester (Mpe, **G**), protochlorophyllide (Pchlide, **H**), chlorophyllide *a* (Chlide *a*, **I**), chlorophyllide *b* (Chlide *b*, **J**), chlorophyll *a* (Chl *a*, **K**), chlorophyll *b* (Chl *b*, **L**), Mg-protoporphyrin IX diester (Mpde, **M**), and heme (**N**) content of control (28 °C) and cold-stressed (18 °C and 12 °C) rice seedlings after 0 h, 0.5 h, 12 h, and 48 h greening. Six day old etiolated seedlings were treated with 18 °C or 12 °C cold stress. Seedlings were harvested at 0 h, 0.5 h, 12 h, and 48 h of greening and their chlorophyll biosynthesis intermediates contents were measured. The relative content of intermediates at 28 °C after 48 h of light exposure was defined as 100%, except Pchlide; the relative content of Pchlide at 0 h was defined as 100% due to its massive accumulation in the dark. The error bars represent standard deviations of three independent biological replicates. Different letters indicate significantly different at *p* < 0.05 according to Duncan’s multiple range tests.

ALA is the first intermediate of chlorophyll biosynthesis. As shown in Figure 3A, light promoted ALA accumulation. After 48 h of light exposure, ALA contents were 19.4% lower at 18 °C and 46.8% lower at 12 °C compared with those at 28 °C. These results indicate that chilling stress inhibited the synthesis of ALA during greening.

PBG content at 28 °C was increased by 14.8%, 104.9%, and 155.2% after 0.5 h, 12 h, and 48 h of light exposure, respectively (Figure 3B), and there was no significant difference of PBG content between chill-treated rice seedlings and the control seedlings after 12 h and 48 h of greening. Urogen III and coprogen III levels were significantly increased at 28 °C during greening (Figure 3C,D). Under low temperatures, the contents of urogen III and coprogen III during greening were higher than those at 28 °C. Proto IX level was gradually decreased after exposure to light, and the rate of decline was accelerated under low temperatures (Figure 3E).

Mg-proto IX is the first intermediate of the Mg branch in the chlorophyll biosynthesis pathway. As shown in Figure 3F, Mg-proto IX increased by 34.5%, 97.5%, and 155.3% after 0.5 h, 12 h, and 48 h of light exposure at 28 °C, respectively. However, the level of Mg-proto IX was significantly decreased during greening under the cold condition. These results demonstrated that chilling stress also inhibited the synthesis of Mg-proto IX during greening. Mg-Proto monomethyl ester (Mpe) content was increased during greening, and was barely influenced by the low temperature (Figure 3G).

In the dark, massive amounts of Pchlide were accumulated, because the protochlorophyllide oxidoreductase (POR) in angiosperms is strictly light-dependent. The etiolated rice seedlings accumulated plenty of Pchlide (Figure 3H). The content of Pchlide was drastically decreased at 28 °C after 0.5 h of light exposure, while Chlide a and Chlide b contents were increased rapidly and then decreased during greening (Figure 3I,J). Compared with 28 °C, the levels of Pchlide were higher but the Chlide a and Chlide b contents were lower in chill-treated groups after 0.5 h and 12 h of light exposure. Obviously, the cold treatment lowered the conversion efficiency of Pchlide to Chlide, especially at 12 °C.

The contents of Chl *a* and Chl *b* were almost undetectable in etiolated seedlings (Figure 3K,L), which were significantly increased at 28 °C during greening. Under low temperatures, the synthesis of Chl *a* and Chl *b* was significantly suppressed during greening, especially at 12 °C. The Chl *a* and Chl *b* contents at 12 °C showed little difference from those in the dark. These results suggested that chilling stress greatly inhibited the synthesis of Chl *a* and Chl *b* during greening.

Mpe can also convert to Mg-protoporphyrin IX diester (Mpde) and then further form Chlide a ester. Mpde contents at both 28 °C and low temperatures were increased during greening (Figure 3M). Heme is the product of the Fe branch, which usually acts as a cofactor in respiration and photosynthesis. Heme contents at different time points had no significant difference during greening (Figure 3N). However, the contents of heme after 48 h of light exposure were decreased by 19.3% and 29.2% at 18 °C and 12 °C, respectively. These results also indicate that chilling stress inhibited the pathway of Fe^2+^ branch during greening.

In summary, the inhibition of chlorophyll biosynthesis under chilling stress may be attributed to inhibited synthesis of ALA and hampered conversion from Pchlide into Chls.

### 2.4. Effect of Chilling Stress on Enzyme Activities in Chlorophyll Biosynthesis

**Figure 4:**
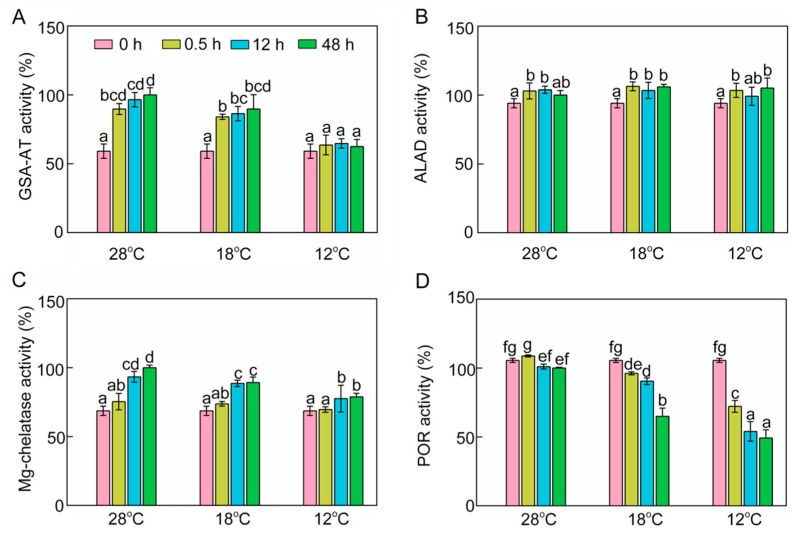
Effects of chilling stress (18 °C and 12 °C) on activities of enzymes involved in chlorophyll biosynthesis. Glutamate-1-semialdehyde transaminase (GSA-AT, **A**), ALA dehydratase (ALAD, **B**), Mg-chelatase (**C**), protochlorophyllide oxidoreductase (POR, **D**) activities of control (28 °C) and chill-stressed (18 °C and 12 °C) rice seedlings after 0 h, 0.5 h, 12 h, and 48 h greening. Six day old etiolated seedlings were treated with 18 °C or 12 °C chilling stress. Seedlings were harvested at 0 h, 0.5 h, 12 h, and 48 h of greening and their activities of enzymes involved in chlorophyll biosynthesis were measured. The activities of enzymes at 28 °C after 48 h of light exposure were defined as 100%. Values are means ± SD from three independent biological replicates. Different letters indicate significant differences according to Duncan’s multiple range tests at *p* < 0.05. Each data point is the average of three replicates. The error bars represent SD.

To further investigate the inhibitory mechanism of chlorophyll biosynthesis, we next examined some key enzymes involved in chlorophyll biosynthesis. Enzymatic activity of glutamate-1-semialdehyde transaminase (GSA-AT), which catalyzes glutamate-1-semialdehyde to ALA, was significantly increased at 28 °C and 18 °C (Figure 4A) during greening. However, low temperature decreased GSA-AT activity, and the activity of GSA-AT at 12 °C had no significant difference from that recorded in the dark (Figure 4A). ALA dehydratase (ALAD) activity was slightly increased after light exposure, and chilling stress had no significant effect on ALAD activity during greening (Figure 4B). Mg-chelatase is a key enzyme which initiates the Mg branch of the chlorophyll biosynthesis pathway. Mg-chelatase activity was increased during greening, and chilling stress had an inhibitory effect on the activity of Mg-chelatase (Figure 4C). POR is a light-dependent enzyme in angiosperms that converts Pchlide to Chlide. POR activity went down gradually during greening (Figure 4D) and chill-treated seedlings showed lower POR activities, especially at 12 °C. In short, the lower ALA content might be attributable to the fact that cold stress inhibited GSA-AT activity, and the inhibition of conversion from Pchlide into Chlide might have been due to the low POR activity under chilling stress.

### 2.5. Effect of Chilling Stress on Transcriptional Expression of Chlorophyll Biosynthetic Genes

**Figure 5:**
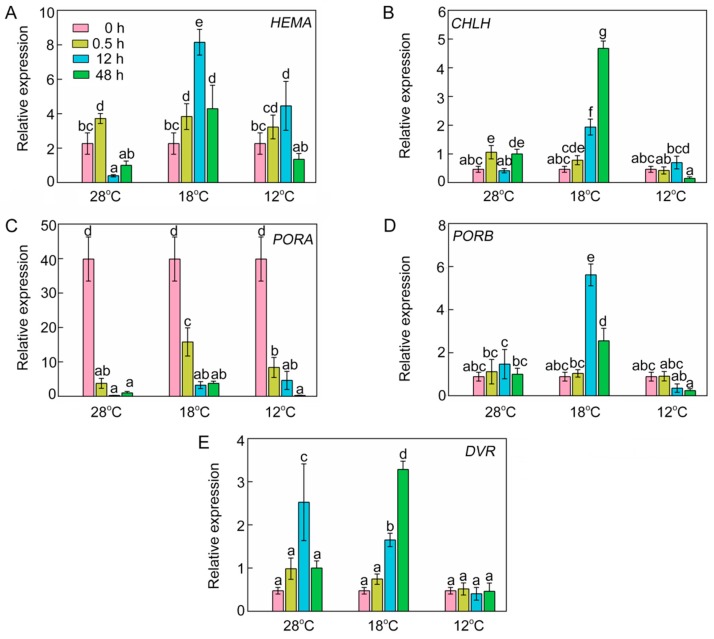
Effect of chilling stress on relative expression of chlorophyll biosynthetic genes *HEMA* (**A**), *CHLH* (**B**), *PORA* (**C**), *PORB* (**D**), and *DVR* (**E**). The expression levels of genes at 28 °C after 48 h of light exposure were set to 1. *OsACTIN1* was used as an internal standard. Values are means ± SD from three independent biological replicates. Different letters indicate significant differences according to Duncan’s multiple range tests (*p < 0.05*).

To decipher the effects of chilling stress on transcription levels of chlorophyll biosynthetic genes, we analyzed the relative expression of four key chlorophyll biosynthetic genes by RT-qPCR. At 28 °C, the expression of *HEMA* was increased initially, and then decreased after 12 h of light exposure (Figure 5A). This phenomenon might have been due to avoidance of the oxidative stress caused by excessive tetrapyrrole intermediate accumulation. However, the chilling stressed rice seedlings accumulated more transcript of *HEMA* after 12 h of light exposure. At 28 °C, the RNA of *CHLH* after 0.5 h of light exposure was slightly higher than that in etiolated seedlings, but the expression of *CHLH* was much higher at 18 °C (Figure 5B). The expression of most chlorophyll biosynthetic genes was inhibited in the dark, but *PORA* transcripts accumulated in the etiolated seedlings but were degraded rapidly upon illumination (Figure 5C). In contrast, *PORB* mRNA level did not fluctuate at 28 °C after illumination. Interestingly, the mRNA level of *PORB* was very high at 18 °C and significantly lowered at 12 °C (Figure 5D). The expression of *DVR* increased at 28 °C and 18 °C after 12 h of light irradiation, but at 12 °C it had no significant difference from that in the dark (Figure 5E). When the temperature was below 18 °C, the expressions of *CHLH*, *PORB*, and *DVR* were severely repressed. Taken together, the repressed expression of chlorophyll biosynthetic genes might be responsible for the inhibition of chlorophyll biosynthesis under low temperatures.

### 2.6. Effect of Chilling Stress on Plastid Proteins during Greening

**Figure 6:**
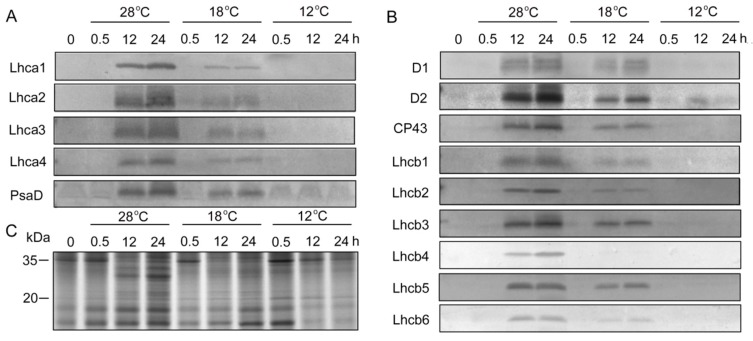
Immunoblot analysis of thylakoid proteins in control and chill-stressed rice seedlings. Six day old etiolated seedlings were treated with 18 °C or 12 °C chilling stress. Thylakoid proteins were isolated from control and chill-stressed seedlings after 0 h, 0.5 h, 12 h, and 48 h of greening. Immunoblot analyses were performed with antibodies specific for representative photosystem I (PSI) (**A**) and photosystem II (PSII) (**B**). The SDS–PAGE of 20 ug plastid protein stained by Coomassie blue (**C**).

To determine whether chilling stress affected plastid protein biosynthesis, immunoblotting analysis was performed (Figure 6). SDS-PAGE showed that the proteins with molecular weight from 20 kDa to 35 kDa under low temperatures were much lower than those at 28 °C after 12 h and 48 h of light irradiation (Figure 6C). In etiolated seedlings, PSI (Lhca1, Lhca2, Lhca3, Lhca4, and PsaD) and PSII (D1, D2, CP43, Lchb1, Lchb2, Lchb3, Lchb4, Lchb5, and Lchb6) proteins were undetected, and large amounts of PSI and PSII proteins were rapidly synthesized at 28 °C during greening (Figure 6A,B). Chilling stress inhibited the accumulation of PSI and PSII proteins during greening (Figure 6A,B, Supplementary Figures S1 and S2), especially at 12 °C, where we hardly detected PSI and PSII proteins.

### 2.7. Effect of Chilling Stress on Chloroplast Biogenesis during Greening

**Figure 7:**
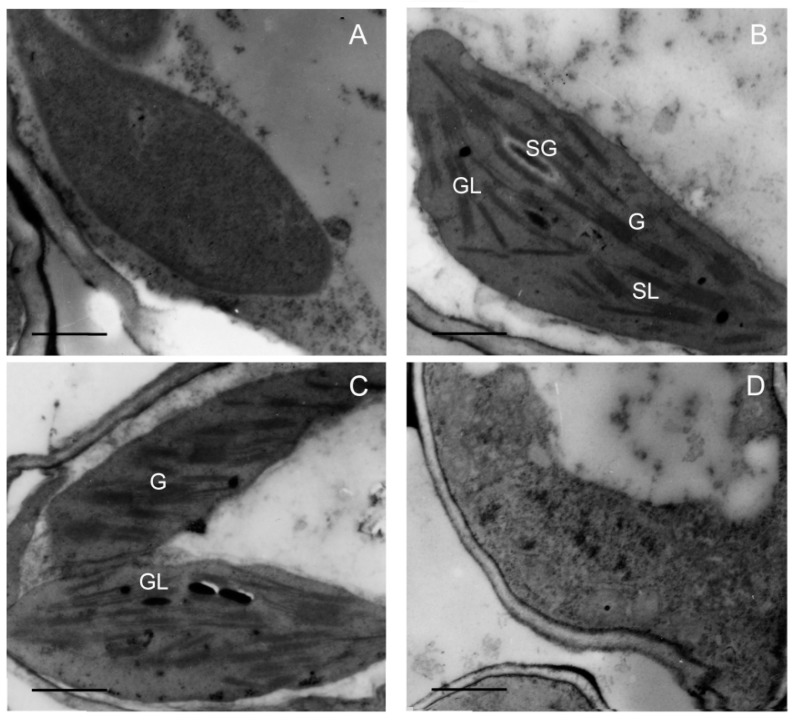
Effect of chilling stress (18 °C and 12 °C) on chloroplast biogenesis of rice seedlings. Plastid ultrastructure of etiolated seedlings (**A**); chloroplast ultrastructure after 48 h of greening under normal temperature (28 °C) condition (**B**); chloroplast ultrastructure after 48 h of greening at 18 °C (**C**); chloroplast ultrastructure after 48 h of greening at 12 °C (**D**). G: granum; SL: stroma lamellae; GL: grana lamellae; SG: starch grain. Bar = 1 μm.

Chloroplast biogenesis normally depends on a stable supply and correct stoichiometry of chlorophyll and photosynthetic proteins during greening. To further investigate the effect of low temperature on plastid development, plastid morphology was analyzed via transmission electron microscopy (TEM). The results showed that the proplastid developed into the etioplast that contains the prolamellar bodies (PLBs) in etiolated seedlings (Figure 7A). When seedlings were illuminated (120 μmol m^−2^ s^−1^) for 48 h at 28 °C, PLBs disappeared and thylakoids formed and grana thylakoids stacked regularly (Figure 7B). Grana stacking was inhibited and thylakoids were much looser at 18 °C (Figure 7C), and no functional thylakoid structure was formed after 48 h of greening at 12 °C (Figure 7D). Taken together, these results suggest that chilling stress inhibited the biogenesis of chloroplast, which might have been due to the lack of chlorophylls and photosynthetic proteins.

**Figure 8:**
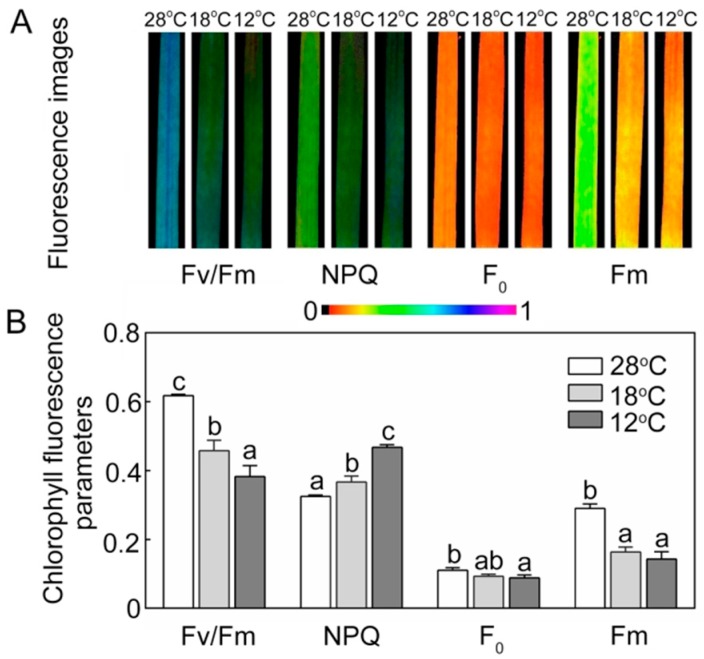
Effect of chilling stress on chlorophyll fluorescence parameters of rice seedlings. The chlorophyll fluorescence images (**A**) and chlorophyll fluorescence parameters (Fv/Fm, NPQ, F_0_, Fm) (**B**) after 48 h of greening. Six day old etiolated seedlings were treated with 18 °C or 12 °C chilling stress. Values are means ± SD from three independent biological replicates. Different letters indicate significant differences according to Duncan’s multiple range tests (*p < 0.05*).

Chlorophyll fluorescence is an important indicator of the work status of chloroplasts. Compared with the seedlings grown under low temperature, the seedlings at 28 °C showed higher quantitative values of maximum PSII yield (Fv/Fm) and lower non-photochemical quenching (NPQ) (Figure 8). Minimal fluorescence yield (F_0_) showed no big fluctuations between 28 °C, 18 °C, and 12 °C treatments, but maximal fluorescence yield (Fm) was significantly lower in the chill-stressed seedlings.

### 2.8. Effect of Chilling Stress on ROS Accumulation, Oxidation, and Electrolyte Leakage during Greening

**Figure 9:**
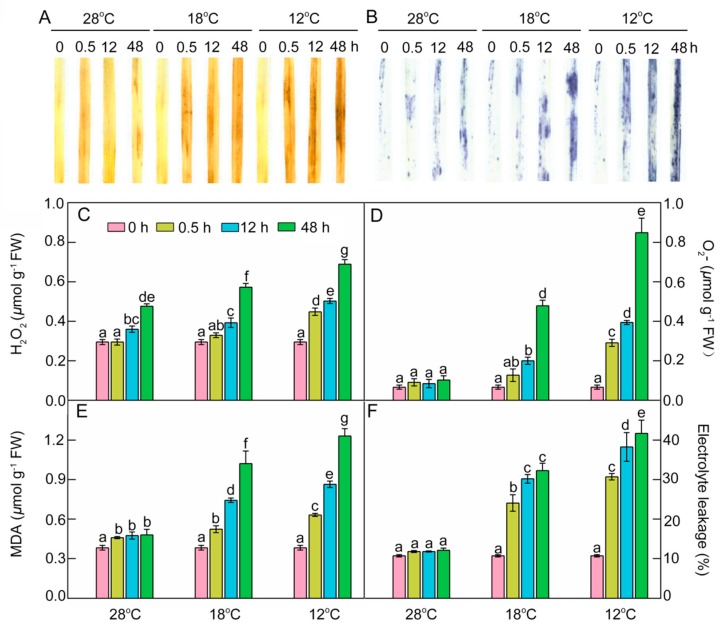
Effects of chill-stressed (18 °C and 12 °C) on H_2_O_2_ (**A**,**C**), O_2_^.−^ (**B**,**D**), MDA content (**E**), and EL (**F**) of rice seedlings. Histochemical detection (**A**,**B**), content of H_2_O_2_ (**C**), O_2_^.−^ (**D**), MDA (**E**), and EL (**F**) of control (28 °C) and chill-stressed (18 °C and 12 °C) rice seedlings after 0 h, 0.5 h, 12 h, and 48 h greening. Six day old etiolated seedlings were treated with 18 °C or 12 °C chilling stress. Seedlings were harvested at 0 h, 0.5 h, 12 h, and 48 h of greening and their ROS levels were measured. Values are means ± SD from three independent biological replicates. Different letters indicate significant differences according to Duncan’s multiple range tests (*p < 0.05*).

As crucial indexes of oxidative damages under chilling stress, the contents of hydrogen peroxide (H_2_O_2_), superoxide anion radical (O_2_^.−^), and malondialdehyde (MDA) were determined. Histochemical detection and quantification analysis showed that H_2_O_2_ increased slightly, but the superoxide anion radical (O_2_^.−^) levels remained almost stable at 28 °C during greening. An ROS burst occurred in chill-treated leaves, especially at 12 °C (Figure 9A–D), indicating that chilling stress could induce ROS accumulation. MDA content and electrolyte leakage (EL) were quantified to examine the lipid peroxidation and the damage to cellular membranes. MDA had a slight increase and EL had no remarkable change at 28 °C during greening, but both MDA and EL significantly increased under low temperatures during greening, especially at 12 °C (Figure 9E,F). These results indicate that chilling stress induced ROS accumulation and caused lipid peroxidation and finally destroyed the integrity of membranes during greening.

### 2.9. Effect of Chilling Stress on Cell Death during Greening

**Figure 10:**
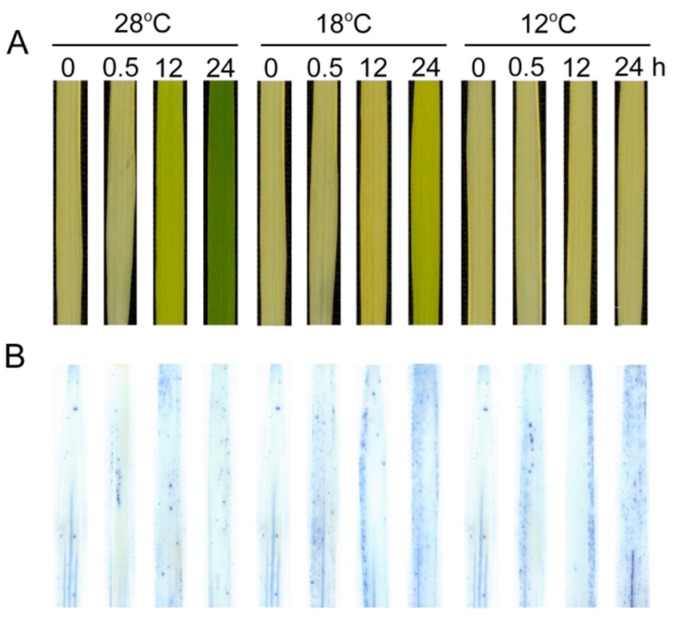
Effect of chilling stress on cell death of rice seedlings. Six day old etiolated seedlings were treated with 18 °C or 12 °C chilling stress. Phenotypes of rice leaves at different time points (**A**); Trypan-blue staining (**B**) of control (28 °C) and chill-stressed (18 °C and 12 °C) leaves after 0 h, 0.5 h, 12 h, and 48 h greening.

We further examined the effect of low temperature on cell death of leaves using Trypan-blue staining (Figure 10). Few cells could be stained at 28 °C, but the number of dead cells significantly increased under low temperatures during greening, especially at 12 °C. These results indicate that the low temperature aggravated cell death during greening.

### 2.10. Effect of Chilling Stress on Epidermis of Rice Leaves during Greening

**Figure 11:**
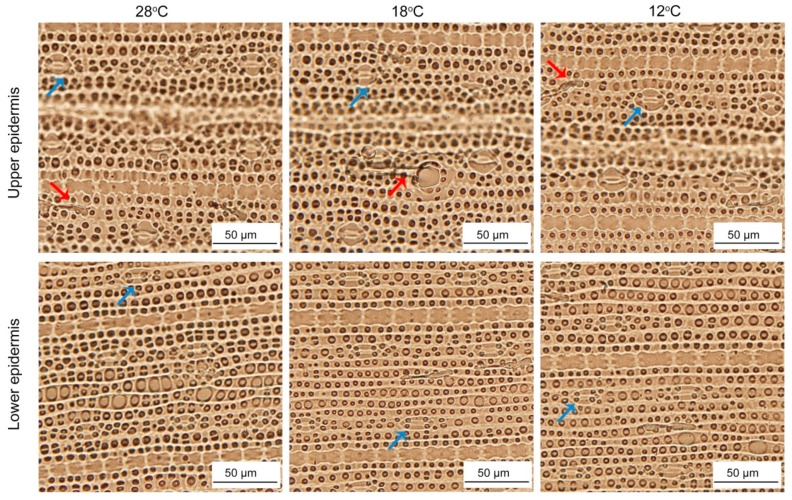
The epidermis cells development of control and chill-stressed rice leaves. Six day old etiolated seedlings were treated with 18 °C or 12 °C chilling stress. Seedlings were harvested at 48 h of greening and their epidermal cells’ characteristics were observed. Red arrows and blue arrows represent trichomes and stoma, respectively.

To investigate whether low temperature affected the epidermal characteristics, we observed the epidermis cells after 48 h of greening. The upper and lower epidermis layers of rice are mainly composed of stomata apparatus and epidermis cells. In addition, there were some trichomes. The shapes and sizes of upper and lower epidermis cells showed no significant difference between the seedlings at 28 °C and low temperatures (Figure 11), while stomata under low temperature were smaller than those at 28 °C (Table 1). Meanwhile, chilling stress had no significant effect on the number of trichomes (Table 1).

## 3. Discussion

Chlorophyll biosynthesis is affected by various biotic and abiotic factors. Previous studies have reported that water and salt stresses lead to the severe inhibition of chlorophyll biosynthesis during de-etiolation [1,30]. Temperature is one of the major environmental factors that can inhibit chlorophyll biosynthesis and chloroplast biogenesis, and thus affect photosynthesis [31,32,33]. Our previous study also showed that cold stress dramatically decreases the net photosynthetic rate, stomatal conductance, intercellular CO_2_ concentration, and water use efficiency in rice seedlings [34]. In this study, our results demonstrated that chlorophyll was significantly increased with the period of light exposure during greening at 28 °C (Figure 2C). However, chlorophyll biosynthesis was obviously inhibited in chill-stressed rice seedlings. Our study further found that ALA synthesis was significantly inhibited in chill-stressed seedlings (Figure 3A). That is to say, the early step of chlorophyll biosynthesis was inhibited by the low temperature, which ultimately led to a significant reduction of chlorophyll and heme contents. Similar changes have also been observed in water- and salt-stressed rice/wheat seedlings during early seedling development [1,30]. Meanwhile, GSA-AT activity was reduced by low temperature during de-etiolation (Figure 4A), suggesting that decreased ALA synthesis in chill-stressed rice seedlings might have been due to the downregulated GSA-AT activity.

In the whole chlorophyll synthesis process, Mg-proto IX and Chlide levels were reduced significantly in stressed seedlings (Figure 3F,I,J), and Mg-chelatase and POR activities were decreased synchronously in chill-stressed seedlings (Figure 4C,D). Declines in Mg-chelatase and POR activities were also observed in water-stressed rice [1]. In plants, Mg-chelatase is composed of three non-identical subunits that are encoded by *CHLI*, *CHLD*, and *CHLH* [35,36]. Previous studies have demonstrated that the mutations of *Chl1* and *Chl9* genes which encode CHLD and CHLI could reduce Mg-chelatase activity, and thus inhibit chlorophyll synthesis [37]. Additionally, ATP is required for the catalytic activity of Mg-chelatase, while ferrochelatase is inhibited by ATP. More Pchlide is allocated to the Mg branch when ATP levels are higher in the light; conversely, the Mg branch is inhibited in the dark [8]. In addition, we found that *CHLH* expression level was much higher at 18 °C than that at 28 °C, but its expression was the lowest at 12 °C. *CHLH* expression in etiolated seedlings was suppressed with lower histone acetylation levels by PIF3, but increased rapidly during greening with higher acetylation levels of histones [38]. Chilling stress might repress histone acetylation levels of *CHLH* at 12 °C. Numerous studies have indicated that post-translational regulation plays an essential role in chlorophyll biosynthesis [39,40,41]. The caseinolytic protease activity counteracts the binding of GluTR-binding protein to assure an appropriate content of GluTR and an adequate ALA synthesis at the post-translational level [42]. This might be why the increased transcriptional levels of *DVR* and *CHLH* under chilling stress were not directly proportional to their corresponding products (Figure 2 and Figure 5). Previous studies have also shown that in water- and chill-stressed rice/cucumber seedlings, Mg-chelatase activity and its gene/protein expression were downregulated [1,43]. The light-dependent POR is a plastid (pro)thylakoid-membrane-associated protein, which binds to NADPH and Pchlide to form a ternary complex in etioplasts [44]. There are three POR isoenzymes in *Arabidopsis thaliana*. *PORA* transcripts accumulate in etiolated seedlings, but the expression of *PORA* is strongly downregulated when exposed to light. The *PORB* transcript can be detected throughout the growth and development of plants, while the expression of *PORC* is induced by light and is predominantly present in fully matured green tissues [8,45]. However, there are only two isoenzymes in rice, namely PORA and PORB [1]. In the present study, POR activity was significantly inhibited in stressed seedlings, especially at 12 °C (Figure 4D). *PORA* transcription decreased dramatically when etiolated rice seedlings were exposed to light at 28 °C, but chill-treated rice seedlings had a relatively high *PORA* mRNA level (Figure 5). Similarly, POR activity and *PORB* transcript abundance were downregulated in water- and chill-stressed rice/cucumber seedlings [1,43]. However, we found that a slightly lowered temperature (18 °C) could significantly increase the expression of *PORB*. As another important tetrapyrrole, heme content showed a 30% drop in seedlings at 12 °C compared to that at 28 °C after 24 h of greening (Figure 3A), which was far less than the declined proportion of chlorophylls, indicating that Chl biosynthesis is more sensitive to low temperatures than heme synthesis. Meanwhile, this result indicated that more tetrapyrrole metabolic intermediates were allocated to the heme branch than to the chlorophyll branch under chilling stress. Thus, the inhibition of chlorophyll biosynthesis under chilling stress might be attributable to the blocked synthesis of ALA and the inhibition of conversion from Pchlide into Chls.

It has been found that the development of thylakoid is inhibited when chlorophyll biosynthesis is reduced [37]. Chlorophyll content was decreased and thylakoid membrane was not stacked in *porB porC* double mutants [46]. These results indicated that the inhibition of chlorophyll biosynthesis affected the biogenesis of chloroplasts, which led to the reduction of granum lamellae and thylakoid membrane proteins. Thus, a stable supply and correct stoichiometry of chlorophyll are necessary for chloroplast biogenesis. In the present study, chloroplast biogenesis was significantly affected by chilling stress (Figure 7C,D). The grana lamellae were disorganized at 18 °C and no grana lamellar structure was formed at 12 °C (Figure 7D). The synthesis of thylakoid membrane protein is of importance to the assembly of the photosystem during greening. Western blotting results showed that thylakoid protein synthesis was obviously inhibited under chilling stress (Figure 6). PSII is considered a primary target of photodamage, and the D1 protein is the most vulnerable component in the PSII reaction center under stress conditions [47]. In this study, the content of D1 was obviously lower under low temperatures, and was almost undetectable at 12 °C. Moreover, low temperatures greatly inhibited the content of the peripheral antenna proteins of PSII, including Lhcb1, Lhcb2, Lhcb3, Lhcb4 (CP29), Lhcb5 (CP26), and Lhcb6 (CP24) and peripheral antenna proteins of PSI, including Lhca1, Lhca2, Lhca3, Lhca4, and PsaD (Figure 6), especially at 12 °C.

Chlorophyll fluorescence analysis has been proven to be a powerful method for obtaining the functional status of PSII [48]. *OsAsr1* rice seedlings have a high value of Fv/Fm, which is correlated with an enhanced cold tolerance [33], suggesting that Fv/Fm could be an indicator of cold tolerance. The decline of Fv/Fm in stressed seedlings may be due to the partial inactivation of PSII reaction centers [49]. In the present study, after 48 h of greening, chill-treated seedlings showed an obvious reduction of Fv/Fm (Figure 8A,B), and the lower Fv/Fm might have been because the low temperature suppressed the assembly and formation of PSII. Meanwhile, the ultrastructural changes of the plastids also indicated that chilling stress affected grana stacking and thylakoid integrity (Figure 6C,D), thereby resulting in a decrease in PSII activity in the stressed plants. Nonphotochemical quenching is a self-protection mechanism in plants. Previous studies have shown that effective heat dissipation in plants can reduce the occurrence of photoinhibition induced by stresses [49]. In this study, the increase of NPQ in stressed seedlings indicated that more excess light energy needed to be dissipated because of the low activity of PSII at low temperatures.

ROS plays double roles under cold stress. On one hand, ROS as a signal can trigger stress-responsive gene expression and the MKK6-MPK3 signaling pathway [26]. On the other hand, excessive ROS in plants can directly induce membrane lipid peroxidation, cell integrity damage, and cell death [50]. PSI and PSII in chloroplasts are the major sources of ROS in plants. Numerous studies have indicated that ROS production increases significantly in plants and the balance of ROS is disturbed under stress conditions [49,51]. Cold signals can be sensed by rice cells through changes in membrane rigidity and osmotic pressure [52]. The membrane rigidity increases under cold stress, resulting in a high electrolyte leakage [53,54]. In accordance with ROS accumulation, the level of lipid peroxidation (MDA) and damage to the cellular membranes were also higher under chilling stress (Figure 9E,F). The increased EL indicated the enhancement of membrane rigidification, which is required for the cold-activated SAMK signaling cascade via cytoskeleton, Ca^2+^ fluxes, and CDPKs [55]. Cold stress initially promotes Ca^2+^ influx into the cytoplasm, which might be controlled by Ca^2+^ channels that are activated by membrane rigidification [56]. Furthermore, the calcium signaling cascade interprets and amplifies the rice-sensing cold signal and subsequently activates the DREB-CRT/DRE pathway, which is important for the cold response [57]. At the same time, our observations showed that leaf cell death was significantly increased under the cold-stress condition during greening (Figure 10). Proline and soluble sugars served as osmoprotectants against oxidative damage, which are also considered indicators to assess the potential cold tolerance of plants [34]. Our previous study indicated that proline and soluble sugar accumulation is enhanced in cold-stressed rice seedlings [34].

Over the past decades, researchers have made extensive efforts to improve cold tolerance in crops, especially in rice. The increasing global food demand, together with rapid population growth and frequent occurrence of chilling forces scientists to speed up and push forward the improvement of rice cold tolerance. ALA synthesis is the rate-limiting step in the whole tetrapyrrole metabolic net, and it is obviously inhibited in chill-treated rice seedlings (Figure 3A). Based on this finding, we propose that application of exogenous ALA or overexpression of *HEMA* or *GSA* gene in plants may overcome inadequate chlorophyll biosynthesis and maintain the structural and functional integrity of chloroplasts, and thus improve cold tolerance. Because tetrapyrrole intermediates are easily activated by light, leading to photooxidative damage, the concentration of exogenous ALA should be applied accurately. Several investigations have recognized that ALA pretreatment enhances plants’ tolerance to chilling by increasing the activities of antioxidant enzymes to eliminate excessive ROS and improving chlorophyll fluorescence and photosynthesis [58,59,60]. Thus, our results suggested the protective role of exogenous ALA and contribute to further illustrate its mechanism. Nevertheless, exogenous ALA increased chlorophyll accumulation in etiolated oilseed rape, but failed to enhance its cold tolerance [61]. Application of exogenous ALA is an exciting field to explore, and might be beneficial for increasing chlorophyll content and improving cold resistance of rice seedlings. Thus, more refined investigations of the effect of exogenous ALA in etiolated seedlings under chilling stress are still required.

Given that transgenic technologies have been developed intensively and almost all chlorophyll biosynthetic genes have been identified in many crops, genetically engineered crops provide a new opportunity to solve the threats from environmental stresses. Thus, genetic modification of chlorophyll biosynthetic genes might be a promising approach for improving plant cold tolerance. A previous study suggested that *CHLG*-over-expressing plants have increased ALA synthetic capacity and increased chelatase activity, indicating that overexpression of *CHLG* could stimulate chlorophyll biosynthesis [62]. Overexpression of *HEMA*, *DVR*, and *CHLG* in plants may be helpful to improve the cold tolerance of rice seedlings during greening by increasing chlorophyll biosynthesis.

## 4. Materials and Methods

### 4.1. Plant Material and Growth Conditions

Rice (*Oryza sativa* L.) cultivar DM You 6188 was used as experimental material, and was purchased from Ya’an seed store. Seeds were sterilized with 3% (*m*/*v*) sodium hypochlorite for 10 min, washed five times with distilled water and soaked in distilled water for 36 h, then placed on moist filter papers with 1/4 strength Hoagland nutrient solution and grown in the dark at 28 °C before chilling treatment. After 6 days, the etiolated seedlings were transferred to vermiculite with 1/4 strength Hoagland nutrient solution in the dark. The seedlings were then exposed to light (120 μmol m^−2^ s^−1^) and transferred to 28 °C, 18 °C, and 12 °C, respectively. The first leaf was used to measure physiological and biochemical parameters at 0 h, 0.5 h, 12 h, and 48 h after exposure to light, and all experiments were repeated at least three times.

### 4.2. Determination of Shoot Dry Weight (DW)

The shoots were collected at 0 h, 0.5 h, 12 h, and 48 h after being exposed to light, washed with tap water and rinsed twice with distilled water, gently wiped with a paper towel, and then oven-dried to a constant weight at 80 °C for DW determination.

### 4.3. Determination of Chlorophyll, Carotenoids and Protein

Chlorophyll and carotenoids were extracted from 0.1 g fresh rice seedlings with 80% acetone. The absorbance of the extract was recorded at 663, 646, and 470 nm according to Lichtenthaler and Wellburn using a spectrophotometer (UV-1750, Shimadzu, Japan) [63]. Protein content was determined by the Bradford method [64].

### 4.4. Determination of Chlorophyll Precursors

δ-Aminolevulinic acid (ALA) was measured according to Dei [65]. Briefly, 0.5 g of fresh leaf was ground in 10 mL of 4% trichloracetic acid with an ice bath, then centrifuged at 18,000× *g* for 15 min. Next, 500 μL of the supernatant was mixed with 2.35 mL of 1 mol/L sodium acetate and 1.5 mL of acetyl-acetone. The mixture was then heated in boiling water for 10 min. After cooling to 25 °C, 2 mL of the mixture was added to 2 mL of Ehrlich-Hg and reacted in the dark for 15 min. The absorption was recorded at 553 nm. The content of ALA was evaluated from the calibration curve prepared from known concentration of ALA.

Porphobilinogen (PBG) was extracted as described by Bogorad [66] with some modifications. Briefly, 0.5 g of fresh leaf was homogenized with 5 mL of extraction solution (0.6 mol/L Tris, 0.1 mol/L EDTA, pH 8.2) in an ice bath and centrifuged for 10 min at 18,000× *g*. Next, 2 mL of the mixture was mixed with 2 mL of Ehrlich-Hg and reacted in the dark for 15 min, with absorbance measured at 553 nm.

Uroporphyrinogen III (urogen III) and coproporphyrinogen III (coprogen III) were assessed according to Bogorad [66] and Rebeiz et al. [67], with some modifications. To determine urogen III content, 1.0 g fresh sample was extracted in an ice bath with 10 mL 0.067 mol/L PBS pH 6.8, then centrifuged for 10 min at 18,000× *g*. Next, 5 mL of the supernatant was mixed 0.25 mL of 1% Na_2_S_2_O_3_. The mixture was illuminated by strong light for 20 min, after which the pH was adjusted to 3.5 with 1 mol/L formic acid. The mixture was extracted three times with 5 mL of ether. The water phase was used to measure the absorbance at 405.5 nm. For analysis of coprogen III, the ether phase was extracted with 0.1 mol/L HCl three times. The HCl phase was used to measure the absorbance at 399.5 nm.

Protoporphyrin IX (Proto IX) was measured based on the method of Rebeiz et al. [67]. Fresh leaves (1.0) were homogenized with extracted solution (acetone: 0.1 mol/L NH_3_·H_2_O, 9:1, *v*/*v*) in an ice bath, then centrifuged for 10 min at 18,000× *g*. Next, 5 mL of the supernatant was mixed with 2 mL n-hexane. The acetone phase was used to record the fluorescence emission spectra at 400, 622, 633, and 640 nm.

Similarly, Mg-protoporphyrin IX (Mg-proto IX), Mg-Proto monomethyl ester (Mpe), Mg-protoporphyrin IX diester (Mpde), protochlorophyllide (Pchlide), and chlorophyllide (Chlide) were determined based on their fluorescence emission spectrums [67]. Heme was extracted and quantified as described by Wilks [68].

### 4.5. Measurement of Chlorophyll Biosynthetic Enzyme Activities

Fresh leaves were collected at 0 h, 0.5 h, 12 h, and 48 h after exposure to light, and immediately grounded with extracting buffers at 4 °C. Glutamate 1-semialdehyde aminotransferase (GSA-AT) activity was analyzed according to Shalygo et al. [62]. ALA dehydratase (ALAD) activity was evaluated as described by Kumar et al. [43]. Mg-chelatase activity was determined based on Yaronskaya et al. [69]. POR activity was measured according to Rebeiz et al. [67].

### 4.6. Isolation of RNA and Quantitative Real-Time PCR

Total RNA was extracted from rice leaves using a Column Plant RNA_OUT_ V1.0 Kit (Tiandz Inc., Beijing, China) according to the manufacturer’s instructions. The first strand of cDNA was synthesized using PrimeScript^TM^ RT reagent Kit with gDNA Eraser (Perfect Real Time) (TaKaRa Bio Inc., Dalian, China), following standard protocol. The quantitative real-time PCR was carried out with the diluted cDNA and SYBR Premix Ex Taq^TM^ II (TaKaRa Bio Inc., Dalian, China) using CFX96 TouchTM Real-Time PCR Detection Systems (Bio-Rad, Chicago, USA), as described previously [70]. The relative expression level of *OsACTIN1* was normalized. The primers used for quantitative real-time PCR are listed in Supplementary Table S1.

### 4.7. Isolation of Thylakoid Proteins and Western Blotting

Thylakoid membrane proteins were isolated as described by Fristedt et al. [71]. Isolated thylakoid membrane protein was separated by SDS-PAGE (5% acrylamide stacking gel + 15% separation gel + 6 M urea) [72]. Western blotting analysis was performed according to Chen et al. [73]. The primary antibodies (all raised in rabbits) including anti-Arabidopsis D1, D2, CP43, LHCb1, LHCb2, LHCb3, LHCb4, LHCb5, LHCb6, LHCa1, LHCa2, LHCa3, and LHCa4, and horseradish-peroxidase-conjugated secondary antibody were purchased from *Agrisera* (Umea, Sweden). The Western blotting signal was detected by a chemiluminescent detection system (ECL, GE Healthcare, Buckinghamshire, UK). The quantification of immunoblots was done with Quantity One software (Bio-Rad, Hercules, CA, United States).

### 4.8. Observation of Transmission Electron Microscopy

Transmission electron microscopy (TEM) analysis of leaves was carried out following a previous method [74]. Leaf tissue was fixed with 3% glutaraldehyde in 0.1 M sodium cacodylate buffer (pH 6.9) at 4 °C overnight, after being washed with phosphate buffer three times. Samples were post-fixed with 2.5% osmium tetroxide, then dehydrated in a gradient solution of alcohol–acetone mixture and embedded in Epon Ultrathin cross sections were cut with an ultramicrotome (Ultracut F-701704, Reichert-Jung, Reichert, Austria), which was then stained with uranyl acetate and observed using a transmission electron microscope (TEM H-9500, Itachi, Tokyo, Japan) operating at 75 kV.

### 4.9. Measurement of Chlorophyll Fluorescence

Chlorophyll fluorescence was imaged using a modulated imaging chlorophyll fluorometer (the Imaging PAM M-Series Chlorophyll Fluorometer System, Heinz-Walz Instruments, Effeltrich, Germany) according to the instructions. The rice leaves were adapted in the dark for 30 min prior to the fluorescence assay, and minimum fluorescence yield (F_0_), maximum fluorescence yield (Fm) and nonphotochemical quenching (NPQ), and maximal quantum yield of PSII photochemistry (Fv/Fm) were then determined according to the method of Zhou et al. [75].

### 4.10. Analysis of Reactive Oxygen Species

Histochemical staining of hydrogen peroxide (H_2_O_2_) and superoxide anion radicals (O_2_^.-^) was performed using 3, 3-diaminobenzidine (DAB) and nitro blue tetrazolium (NBT), respectively [76]. Leaf tissue was immersed in NBT (1 mg/mL) solution for 2 h or in DAB (0.5 mg/mL) solution for 12 h in the dark. The stained leaves were decolorized in boiling ethanol (90%, *v*/*v*) for 2 h. H_2_O_2_ was quantitated according to Velikova et al. [77] and calculated using a standard curve of H_2_O_2_ reagent. The quantification of O_2_^.-^ was determined as described by Nahar et al. [78] and calculated using a standard curve of a NaNO_2_ reagent.

### 4.11. Determination of Malondialdehyde (MDA) and Electrolyte Leakage (EL)

The level of membrane lipid peroxidation was estimated by MDA content, which was determined by thiobarbituric acid (TBA) assay [78]. Fresh leaves (0.5 g) were homogenized in 5 mL of 5% (*m*/*v*) trichloroacetic acid (TCA) and centrifuged at 4 °C for 10 min at 8000× *g*. Next, 2 mL of 0.5% TCA containing 0.67% TBA was added to 2 mL of the supernatant. The mixture was incubated at 95 °C for 30 min and then instantly cooled on ice and centrifuged at 4 °C for 10 min at 5000× *g*. The absorbance of the supernatant was recorded at 450, 532 nm, and 600 nm.

EL of rice leaves was determined using an electrical conductivity meter (DDS-309+, Chengdu, China) following Ning et al. [79]. Relative EL was expressed as the ratio of initial conductivity to the conductivity after the samples were boiled for 15 min to achieve 100% electrolyte leakage.

### 4.12. Trypan-Blue Staining

Dead cells were visually detected using a Trypan-blue staining method as described by Liang et al. [80] with some modifications. Leaves were stained with lactophenol–Trypan blue solution for 20 min under vacuum conditions. The stained leaves were then decolorized in boiling ethanol (90%, *v*/*v*) for 2 h. Samples were equilibrated with 70% glycerol for scanning.

### 4.13. Characteristics of Epidermis Cell and Stomata

The upper and lower epidermis layers of rice leaves after 48 h of greening were observed using light microscopy by transparent nail polish imprint method [81]. The epidermis cells’ characteristics and stomatal characteristics, including stomatal length, width, size, and density, were observed via Olympus fluorescence microscopy (DP71) (40 × 10) and measured and counted by an image analysis system (Image-Pro Plus 6.3). Five microscope fields were randomly selected in each slide. Each treatment group was repeated 50 times to measure all the characteristic parameters under the microscope.

### 4.14. Statistical Analysis

All experiments were repeated at least three times, and mean values are presented with standard deviation (SD). The data analysis was performed using IBM SPSS Statistics. Duncan’s multiplication range test was used for comparison among different treatments. The difference was considered to be statistically significant when *p* < 0.05.

## 5. Conclusions

In summary, our results showed that chilling stress induced ROS accumulation, leading to lipid peroxidation and cell death during greening. In particular, our data highlighted the detailed regulation of photosynthetic pigments under chilling stress during greening, suggesting that the inhibition of chlorophyll biosynthesis may be attributable to the blocked synthesis of ALA and the inhibition of conversion from Pchlide into chlorophylls. In this case, we have proposed that application of ALA or overexpression of *HEMA*, *DVR*, and *CHLG* may be good for increasing chlorophyll biosynthesis and improving cold resistance of rice seedlings during greening.

## Figures and Tables

**Table 1:** Effect of chilling stress on stomatal characteristics and trichomes number of rice seedlings.

Type of Leaf		Stomata Length (μm)	Stomata Width (μm)	Stomata Size (μm^2^)	Stomata Density (No·mm^−2^)	Trichomes (Per Unit leaf)
28 °C	Upper epidermis	27.50 ± 3.28a	18.40 ± 1.23a	384.32 ± 24.75a	195.51 ± 14.97bc	49.61 ± 10.63a
	Lower epidermis	21.92 ± 1.73c	15.60 ± 1.34b	307.98 ± 20.86b	210.94 ± 25.69a	48.51 ± 6.91a
18 °C	Upper epidermis	23.43 ± 1.44b	15.32 ± 1.74b	303.59 ± 20.76bc	193.67 ± 16.56bc	52.18 ± 10.08a
	Lower epidermis	21.80 ± 1.52c	13.66 ± 1.08c	294.31 ± 16.00c	206.53 ± 30.24ab	51.45 ± 10.11a
12 °C	Upper epidermis	23.19 ± 1.62b	13.57 ± 0.89c	299.30 ± 17.94bc	184.12 ± 22.68c	51.08 ± 9.38a
	Lower epidermis	22.62 ± 1.62c	13.63 ± 0.91c	270.20 ± 26.74d	195.88 ± 14.94bc	51.45 ± 5.33a

The values are expressed as mean ± SD (*n* = 50); different letters represent significant difference (*p < 0.05*).

## Supplementary Materials

Supplementary materials can be found at https://www.mdpi.com/1422-0067/21/4/1390/s1. Figure S1: Relative content of PSI proteins in control and chill-stressed rice seedlings. Figure S2: Relative content of PSI proteins in control and chill-stressed rice seedlings. Table S1: The primers used for quantitative real-time PCR.
