# Supplementary material for: Effect of Low Temperature on Chlorophyll Biosynthesis and Chloroplast Biogenesis of Rice Seedlings during Greening
Source: Int J Mol Sci. 2020 Feb 19;21(4):1390. doi: 10.3390/ijms21041390 (PMC7073065; doi:10.3390/ijms21041390)
Supplement: Supplementary file 1 [file ijms-21-01390-s001.pdf]

Supplementary Material

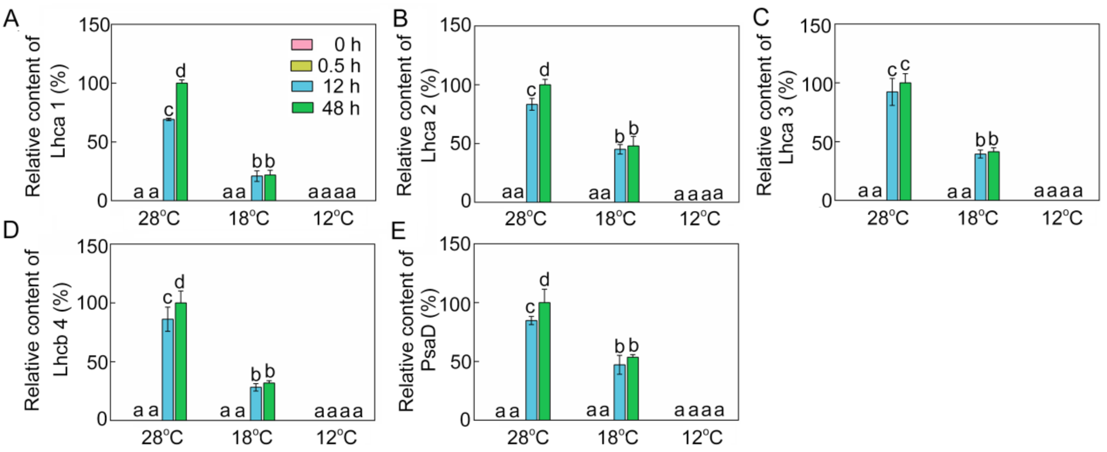

Figure S1. Relative content of PSI proteins in control and chill-stressed rice seedlings, Lhca1 (**A**), Lhca2 (**B**), Lhca3 (**C**), Lhca4 (**D**), PsaD (**E**). Six-day-old etiolated seedlings were treated with 18 °C or 12 °C chilling stress. Thylakoid proteins were isolated from control and chill-stressed seedlings after 0 h, 0.5 h, 12 h and 48 h of greening. The relative content of proteins at 28 °C after 48 h of light exposure was defined as 100%. Loading was according to an equal amount of proteins. Each data point is the average of three replicates. The error bars represent SD. Different letters indicate significant differences according to Duncan's multiple range tests ($p < 0.05$).

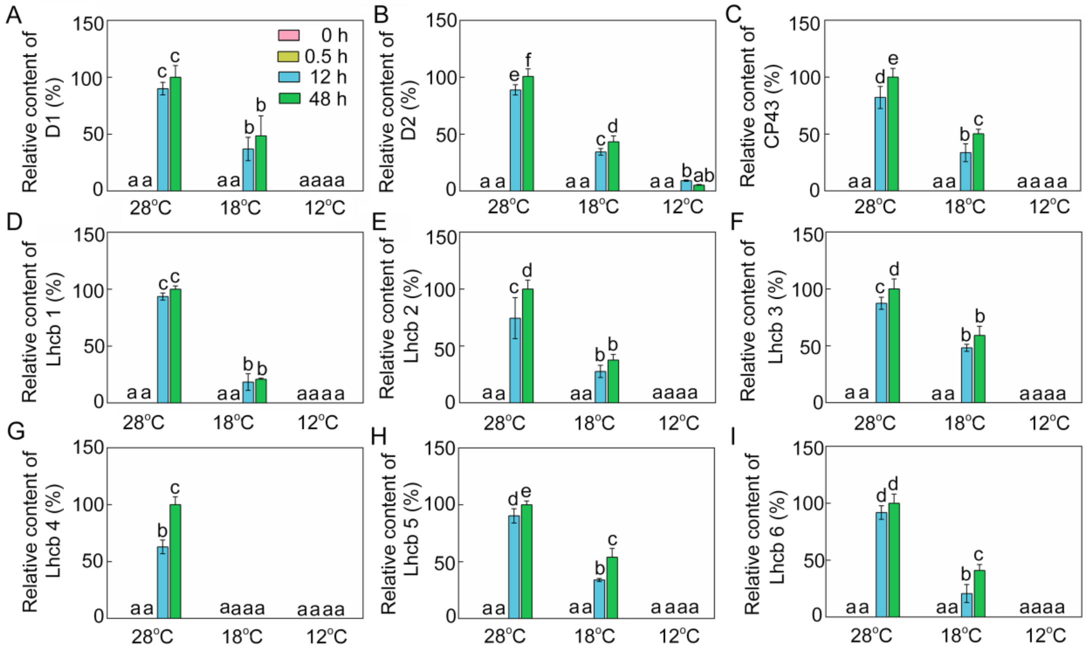

Figure S2. Relative content of PSI proteins in control and chill-stressed rice seedlings, D1 (**A**), D2 (**B**), CP43 (**C**), Lhcb1 (**D**), Lhcb2 (**E**), Lhcb3 (**F**), Lhcb4 (**G**),Lhcb5 (**H**), Lhcb6 (**I**). Six-day-old

etiolated seedlings were treated with 18 °C or 12 °C chilling stress. Thylakoid proteins were isolated from control and chill-stressed seedlings after 0 h, 0.5 h, 12 h and 48 h of greening. The relative content of proteins at 28 °C after 48 h of light exposure was defined as 100%. Loading was according to an equal amount of proteins. Each data point is the average of three replicates. The error bars represent SD. Different letters indicate significant differences according to Duncan's multiple range tests ($p < 0.05$).

Table S1. The primers used for quantitative real-time PCR.

**Gene**	**Forward Primer (5'–3')**	**Reverse Primer (5'–3')**
OsHEMA	CGCTATTTCTGATGCTATGGGT	TCTTGGGTGATGATTGTTTGG
OsCHLH	AACTGGATGAGCCAGAAGAGA	AAATGCAAAAGACTTGCGACT
OsPORA	ATGGCTCTCCAAGTTCAG	TGGCTCACGCTAAGGAAC
OsPORB	CCGCAAGGAGGGAGCGGTG	CCCTCTTGGTGCTAAGGCCG
OsDVR	AGCCCAGGTTCATCAAGGT	TGATCACCCTCTCGAAGAACT
